# Previous binaural experience supports compensatory strategies in hearing-impaired children’s auditory horizontal localization

**DOI:** 10.1371/journal.pone.0312073

**Published:** 2024-12-05

**Authors:** Andrea Gulli, Federico Fontana, Alessandro Aruffo, Eva Orzan, Enrico Muzzi

**Affiliations:** 1 Department of Engineering and Management, University of Padua, Padua, Italy; 2 HCI Lab, Department of Mathematics, Computer Science and Physics, University of Udine, Udine, Italy; 3 Otorhinolaryngology and Audiology, Institute for Maternal and Child Health IRCCS “Burlo Garofolo”, Trieste, Italy; Indiana University, UNITED STATES OF AMERICA

## Abstract

This study investigates auditory localization in children with a diagnosis of hearing impairment rehabilitated with bilateral cochlear implants or hearing aids. Localization accuracy in the anterior horizontal field and its distribution along the angular position of the source were analyzed. Participants performed a localization task in a virtual environment where they could move their heads freely and were asked to point to an invisible sound source. The source was rendered using a loudspeaker set arranged as a semi-circular array in the horizontal plane. The participants’ head positions were tracked while their hands pointed to the auditory target; the preferred listening position and the onset of active strategies involving head movement were extracted. A significant correlation was found between age and localization accuracy and age and head movement in children with bilateral hearing aids. Investigating conditions where no, one, or both hearing devices were turned off, it was found that asymmetrical hearing caused the largest errors. Under this specific condition, head movement was used erratically by children with bilateral cochlear implants who focused on postures maximizing sound intensity at the more sensitive ear. Conversely, those with a consolidated binaural hearing experience could use dynamic cues even if one hearing aid was turned off. This finding may have implications for the clinical evaluation and rehabilitation of individuals with hearing impairments.

## Introduction

Spatial hearing is an essential component of auditory scene analysis, primarily aimed at localizing acoustic events, segregating auditory streams, and orienting multisensory attention [1]. Sound localization is based on sound directionality and distance estimation derived from the binaural processing of loudness, time delay, phase, and spectral cues. The most important cues are the interaural time difference (ITD) and the interaural level difference (ILD), determined by the spatial separation of the two ears [2]. Useful information for localization can also be extracted from monaural cues, such as time and level differences between individual spectral components. Providing identical signals at both ears proved that monaural cues help define the anterior and posterior sectors of the midplane, the elevation angle, and the distance of the auditory event [3]. Slight head and body movements, taking place even when trying to keep still while listening, effectively resolve front-back confusion [4] and improve the precision of auditory spatial recognition [5].

Spontaneous head movements in response to auditory cues enhance horizontal localization by turning static binaural cues into dynamic information [6]. This dynamic exploration of acoustic space has been defined as active listening [7]. Nevertheless, a common principle underlying the different head dynamics observed during sound localization has yet to be found [8]. Head movement patterns differ among individuals, suggesting that every active localization strategy entails subjective rotational and translational movements involving the torso, head, and eyes [9]. Therefore, monitoring head movements during acoustic localization in different populations is not obvious. Normal-hearing (NH) individuals in a simulated asymmetric hearing loss condition possibly exploit their binaural experience [10] and behave differently from a hearing-impaired (HI) population. An elderly population, NH or HI alike, can localize a sound source more accurately through active listening than children [11].

Hearing loss impacts every facet of auditory perception, including the ability to accurately determine the direction of sound sources [12]. Spatial hearing impairment harms awareness of one’s surroundings, personal safety, and social interaction. Binaural restoration using bilateral cochlear implants (CIs) offers a better quality of hearing than unilateral implantation [13]. The recent advances in sound processing technologies feature elements of spatial rendering for auditory impaired individuals [14]. However, in many cases, acoustic or electric auditory stimulation rehabilitation fails to restore the cues relevant to spatial hearing [5]. Bilateral hearing aids (HAs) should provide interaural level and time cues. Nevertheless, inconsistencies in localization have often been found in patients, suggesting that the benefit, although significant, is usually poorer than predicted [15]. On the other hand, CIs distort ITD cues, so patients must rely on ILD cues for sound localization [16]. Furthermore, since several CI microphones are positioned behind the pinna, most of the amplitude and frequency cues conveyed by the outer ear are lost. The resulting lack of monaural cues deprives infants and children of the timely development of their acoustic localization skills [17].

Restoring normal hearing is critical in children because hearing loss can harm a regular speech and language development. HAs [18] and CIs [19] proved effective in improving speech perception and production. Evolving traditional clinical assessments based on pure tone audiometry [20] is crucial, though. Assessing the benefits of assistive hearing devices during one’s everyday routine tasks can provide future directions for technological developments.

Previous studies demonstrated that children with bilateral cochlear implants are sensitive to ILDs, possibly due to monaural level cues [11]. Children who are deaf from birth have weak or absent sensitivity to ITDs. Conversely, children who could rely on previous listening experience are sensitive to these cues [11]. Another study confirmed that better binaural fusion is associated with an extended hearing experience before cochlear implantation. These observations highlight the importance of enabling auditory perception as an essential component in children’s development [21]. On the other hand, binaural localization in HI children who wear a device during their auditory development is still partially unexplored, and the role of head movement is yet to be understood.

The primary objective of this study was to examine how two pediatric populations—one using bilateral HAs and the other using CIs—localize sound in the horizontal plane. We assessed their spatial hearing abilities based on performance, active listening, and preferred listening position, then compared such two populations with each other and with NH listeners. The second objective was to estimate the potential of bilateral assistive devices to induce binaural sensitivity. We analyzed the effect of deactivating one or both devices by examining the residual localization ability as a function of the sound source position.

The correlation between variables also provided insights into potential localization strategies. Previous research has shown that NH listeners increase their head movements during a localization task with simulated asymmetric hearing loss [22]. We aimed to determine whether this behavior also occurs in listeners using HAs and CIs, either as a response to asymmetric hearing conditions, or rather as a characteristic response of individuals with a consolidated binaural hearing experience. Correlating their head angular positions and movements with localization accuracy gave insights into their residual binaural ability. This helped us better understand the role of head position and movements in HA and CI populations. Furthermore, a correlation analysis between localization abilities and age provided information on one’s ability to consolidate spatial hearing through experience.

The task was performed in a mixed (i.e., real and virtual) environment. Listeners wearing a head-mounted display (HMD) used a virtual laser beam to point to a sound source position reproduced by a loudspeaker array. This design choice was supported by research that found no significant difference in the horizontal localization accuracy of NH listeners in real environments or virtual replicas [23]. Head movements and hand pointing were simultaneously tracked to collect temporal data about head motor activity during the localization task. Based on a setup and methodology already tested on an NH population [24], we assessed whether and how head dynamics and orientation could compensate for adverse listening conditions. We turned off one hearing device causing asymmetric listening, or both devices when possible, thus restoring native listening. This experimental design established a valid procedure for observing the onset of binaural sensitivity.

A preliminary analysis [25] of the results from this procedure has been largely reformulated here. We interpreted head movements in such adverse hearing conditions as an indicator of subjective willingness to restore binaural cues. We expected that children with no motor disorders put diverse compensation strategies into action, depending on their residual localization ability, once their bilateral hearing devices are switched off on either or both ears. We aimed to compare their active listening against NH individuals [26]. We hypothesized bilateral hearing devices helped HI children with spatial hearing, but they failed to evoke ITD cues. The sensitivity to these cues was instead the result of a consolidated hearing experience. More speculatively, classifying HI children’s compensatory strategies may inform future rehabilitation and training for localization, speech-in-noise detection, and spatial sound listening [27]. For example, knowing that ITD sensitivity can be neither found nor enabled in a particular population may lead to rehabilitation programs focusing on monaural localization using adequate acoustic stimuli.

## Materials and methods

The study was approved by the Institutional Review Board of the Institute for Maternal and Child Health IRCCS “Burlo Garofolo” (Trieste, Italy) under the project “Ricerca Corrente 17/23”. Informed consent has been obtained for each participant from their parents.

### Participants

Twenty-two HI children (13 males and 9 females, mean age *μ* = 10.45 years, standard deviation *σ* = 3.13 years) participated in the experiment. Nine were CI listeners, and thirteen were HA listeners. Children were affected by non-syndromic hearing loss (“GEN NO SDR”) in 8 cases (6 GJB2 gene mutations, 2 other gene mutations), syndromic hearing loss (“SDR”) in 4 (2 Usher syndromes, 1 chromosomal instability, 1 Waardenburg syndrome), and 1 enlarged vestibular aqueduct (inner ear malformation, “IEM”). Other causes of hearing loss (“Other”) were congenital cytomegalovirus infection in 2 cases, chemotherapy with platinum derivatives for neuroblastoma in 2, preterm delivery in 1, and prolonged neonatal intensive care unit stay in 1 case. The cause was not identified (“ND”) in 3 cases. The two populations were similar in age (*μ* = 10.3 years, *σ* = 3.0 years for the CI listeners and *μ* = 10.5 years, *σ* = 3.2 years for the HA listeners), but they differed substantially in terms of interaural experience of their devices (*μ* = 2.4 years, *σ* = 2.9 years for the CI listeners and *μ* = 0.1 years, *σ* = 0.3 years for the HA listeners). All participants confirmed verbally that they were right-handed and had no diagnosis of motor impairment. Recruitment started on June 17th, 2022, and ended on January 19th, 2023. Anonymized data became accessible to the authors on January 22nd, 2023. Table 1 displays the collected participants’ data.

**Table 1:** Age, cause of hearing impairment (GEN NO SDR: Non-syndromic hearing loss; SDR: Syndromic hearing loss; IEM: Inner ear malformation; ND: Not identified, other: Other causes), ear devices, and years of experience with the device for each child.

#	Age (years)	Cause	Devices		Experience (years)	
			Left	Right	Left	Right
1	7	Other	Cochlear CI532 Kanso 2	Cochlear CI532 Kanso 2	4	5
2	14	GEN NO SDR	Cochlear CI532 CP1000	Cochlear CI532 CP910	5	5
3	14	GEN NO SDR	Cochlear CI532 CP1000	Cochlear CI532 CP1000	12	2
4	8	GEN NO SDR	Cochlear CI532 Kanso	Cochlear CI512 CP910	5	7
5	6	IEM	MED-EL SYNCHRONY SONNET	MED-EL SYNCHRONY SONNET	5	5
6	9	SDR	Cochlear CI532 CP910	Cochlear CI532 CP910	6	8
7	12	ND	Cochlear CI24RE (CA) CP1000	Cochlear CI512 CE CP910	10	6
8	14	Other	Cochlear CI512 CP1000	Cochlear CI24RE (CA) CP1000	7	8
9	9	GEN NO SDR	Cochlear CI512 CP910	Cochlear CI512 CP910	6	8
10	15	SDR	HA Beltone Legend 17	HA Beltone Legend 17	6	6
11	9	Other	HA Phonak Sky B70-P	HA Phonak Sky B70-P	4	4
12	8	SDR	HA Ampli EnergyB 3 P R+	HA Ampli EnergyB 3 P R+	5	5
13	13	ND	HA Beltone Legend 17	HA Beltone Legend 17	4	4
14	17	GEN NO SDR	HA ReSound Linx 3D 767	HA ReSound Linx 3D 767	7	7
15	12	SDR	HA Beltone Legend 17	HA Beltone Legend 17	4	4
16	13	Other	HA ReSound Linx 3D 767	HA ReSound Linx 3D 767	8	7
17	6	GEN NO SDR	HA ReSound Linx 3D 767	HA ReSound Linx 3D 767	3	3
18	9	Other	HA Oticon Opn Play 1 MiniRITE	HA Oticon Opn Play 1 MiniRITE	8	8
19	7	ND	HA Oticon Ria2 Pro M.R.	HA Oticon Ria2 Pro M.R.	4	4
20	11	GEN NO SDR	HA Resound Linx 3D 767	HA Resound Linx 3D 767	4	4
21	7	Other	HA Phonak Sky B70-P	HA Phonak Sky B70-P	1	1
22	10	GEN NO SDR	HA Phonak Audéo Paradise 90	HA Phonak Audéo Paradise 90	6	6

### Setup

The acoustic reproduction system consisted of 13 Seeburg i4 loudspeakers (SEEBURG acoustic line GmbH) driven by a Sonible d:24 multi-channel amplifier (Sonible GmbH). These loudspeakers were arranged to form a semi-circular array with a radius equal to 1.4 m in a small enclosure measuring 3×2.6 m, having a 60 dB-reverberation time of 200 ms. With this array, 13 sound sources radiating from equally-spaced horizontal angles of arrival were reproduced across an egocentric scene spanning between −90° and + 90°, with angles equal to 15° between each pair of adjacent loudspeakers.

A three-dimensional (3D) virtual environment (VE) was developed in the Unity3D programming environment. An Oculus Quest 2 HMD, including the Oculus Touch hand controllers (Meta Platforms Technologies Ireland Limited, Dublin, Ireland), was employed to reproduce the visual scene. The HMD tracks the 3D position of the head with submillimeter precision [28]. The four-valued quaternion representing the orientation of the head in the 3D scene had a precision of ±1° at 20 Hz sampling rate [29]. The data from the HMD were received via the mqtt protocol [30] running on a 2.4 GHz Wi-Fi connection by an mqtt broker as a Docker container (Docker, Inc.). From here, data were sent to a custom client app, allowing the experimenter to monitor head tracking and hand pointing and to check whether the connection between the Oculus and the computer was constantly up and running. A Max (Cycling ’74) real-time sound synthesis patch running on the same computer was used to reproduce the auditory scene at runtime. Sounds with 16-bit resolution at 44.1 kHz sampling frequency were sent to the loudspeakers by a MADIface USB 2.0 Audio Interface (RME GmbH).

A seat whose height could be adjusted by the experimenter was placed on the focal point of the semi-circular array so that the head was distant 1.4 m from the speakers at elevation zero. The experimenter aligned each virtual source to the corresponding loudspeaker by aiming at each speaker from the seat and then reading the angle displayed by the client app. This angle defined the *target* angle. The angular resolution of the pointer was set to 1 degree, based on the Oculus controller accuracy rotation found in the literature [31].

### Stimuli

The acoustic stimulus consisted of pulsated pink noise, with each burst lasting 200 ms and completed with a 100 ms linear onset and a 100 ms linear decay. Adjacent noise bursts were separated by 200 ms of silence. This acoustic stimulus has been selected because normal listeners localize it easily; it has a rich broadband spectrum [32]. Furthermore, it provides a periodic temporal envelope enabling listeners to capture the binaural cues relevant to spatial hearing. The pulsated stimulus lasted until a participant produced a response. It was presented at a sound pressure level (SPL) equal to 65 ± 1 dB, measured with a calibrated meter (XL2 Sound Level Meter, NTi Audio). SPL was measured during setup by aligning the meter to the experimenter’s external ear while he was seated in the test focal point. The measurement was repeated for each speaker on both ears.

### Procedure

Before each session, the seat’s height was adjusted to align the loudspeakers at the participant’s ear level. Then, participants were instructed to use one controller with their dominant hand to point toward the sound source position. At this point, they were invited to sit and wear the HMD. The interpupillary distance was adjusted, and the real and virtual worlds were aligned in the HMD through a spatial calibration procedure. Calibration was performed by asking each participant to point to specific visual markers occupying a playground until the system recorded every hit to a marker to mismatch with the corresponding loudspeaker by less than 1°. The resulting calibration was loaded through the Oculus Guardian system.

**Fig 1:**
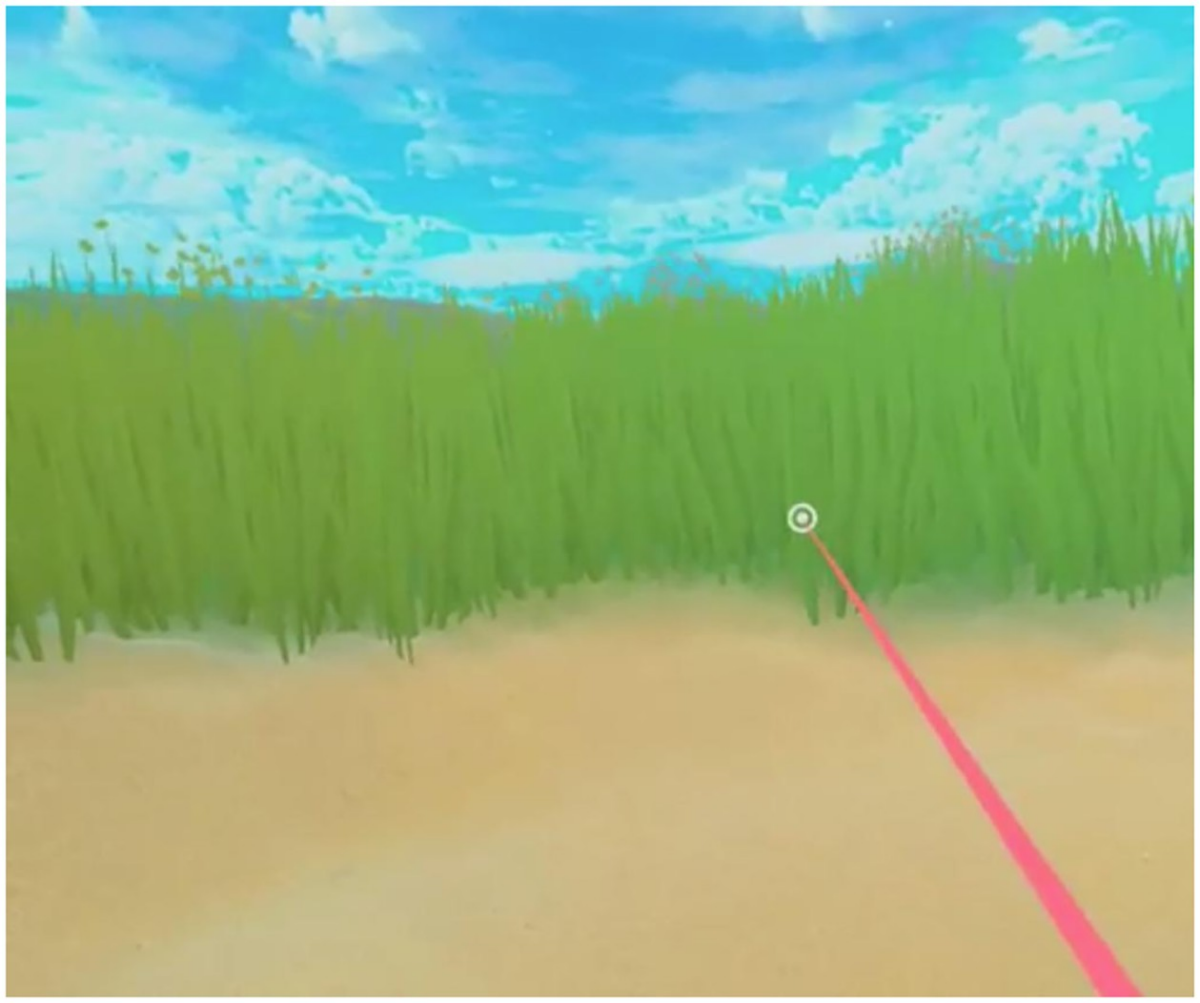
The 3D scene with the beam pointing to a guessed sound source position.

During the task, participants were immersed in a VE consisting of a homogeneous landscape free of any absolute azimuth reference (see Fig 1). They were listening to sounds coming from the loudspeakers and were in a condition to point to a guessed sound source position with the controller in their hand. A beam was displayed to give participants visual feedback about the pointing direction. Participants were not instructed to respond as soon as they heard a sound nor informed that the stimuli came only from the frontal hemifield.

Before a test session started, participants completed a brief training session of five trials. They were invited to take the time they needed to produce a response. Each trial began when one loudspeaker started to reproduce a stimulus; concurrently, the system started to track the participant’s head movement. It finished when a participant pulled the Oculus Touch trigger. At this moment, the acoustic stimulus stopped. The system recorded the guessed angular position and the trajectory of the participant’s head during the trial. After pausing for one second in silence, a new trial began.

Before a test session started, participants completed a brief training session of five trials. They were invited to take the time they needed to produce a response. Each trial began when one loudspeaker started to reproduce a stimulus; concurrently, the system started to track the participant’s head movement. It finished when a participant pulled the Oculus Touch trigger. At this moment, the acoustic stimulus stopped; the system recorded the guessed angular position and the trajectory of the participant’s head during the trial. After pausing for one second without any acoustic stimuli, a new trial began.

### Conditions

Each session included multiple test conditions: up to four for the HA listeners and three for the CI listeners. In each condition, the stimulus was presented 5 times from each loudspeaker position across a sequence of 13 × 5 = 65 trials. The sequence was constrained to equate the number of target position shifts alternating leftward and rightward. Every participant received a randomly rotated version of this constrained sequence in such a way that our listeners completed a random module of a series of angular arcs in a test session. To this end, the angular position forming the sequence tail was pasted to the sequence head, and later, the first trial was removed from the analysis. We deliberately chose not to reset the participant’s head to a starting position after each trial since the literature reports that interruptions decrease performance in individuals who are cognitively engaged in a demanding task [33]. Considering the age of our population we favored engagement, by letting participants keep focus on the pointing task.

Each participant performed the task first with both devices turned on (“ON”), then with one device turned on and one off (“L” for the left device turned on, or “R” for the right device turned on, respectively) by randomly starting with either the left or right ear, and finally with both devices turned off (“NO”). The ON condition was presented first during each test session because it provided an everyday listening context to which participants were accustomed and confident. The NO condition was omitted if a patient’s pure tone average threshold in the frequency range [0.5-4] kHz was above the stimulus level used for the test. For this reason, only nine HA listeners completed the NO condition. Five HA listeners could not complete the L and R conditions either since their session had to be stopped immediately as they reported annoyance or fatigue to the experimenter. In the end, four HA listeners completed the whole session in all four conditions; four completed only the ON, L, and R conditions, and five completed only the ON and NO conditions. Conversely, CI listeners completed the ON, L, and R conditions. We analyzed only sessions including the complete set of 65 trials, except seven sessions that were completed by CI listeners (two in both the R and L conditions, one in the R condition, one in the L condition, and one in the ON condition), each missing one trial that was not recorded due to a technical problem.

### Data analysis

From the 3D array of the positions and the 4D quaternion, we computed the difference between the target angle and head orientation angle when a response was produced (*head rotation*) and the total distance covered by the head during each trial (*head distance*). The latter has already been used to measure head dynamism in studies examining spontaneous actions during music listening [34]. The *signed error* was computed as the difference between the target and the pointed angle [35]. From it, we computed the *unsigned error* as the absolute value of the signed error. While the signed error indicated angular bias across repeated trials (e.g., the tendency to shift leftward or rightward), the unsigned error quantified overall accuracy. In the following, we will name the signed error as bias and the unsigned error as accuracy. Although the unsigned error is continuous by nature, its measurement to the sexagesimal degree had to be analyzed with data bins 1 degree apart from each other due to the precision of the Oculus.

The experiment had a mixed design, where the factors were the two populations with HAs and CIs and conditions, four for the former population and three for the latter. No participant with CIs had a hearing threshold sufficient for attending the NO condition; hence, seven *subsets* were analyzed. As mentioned before, not all participants performed the test in every condition. The mean *μ* and standard deviation *σ* of the unsigned error were computed for each subset. Trials resulting in unsigned errors larger than three standard deviations above the mean deviation per target angle were considered outliers and removed from the respective subset [36]. Exclusion of the outliers is a common procedure in sound localization studies [23, 35]. In our case, the outliers were finally 79 out of 4218 trials, i.e., 1.87%. A wide variance in the data was noted during the training sessions, especially in terms of localization accuracy by the CI listeners. As in other studies [16], this variance was mapped on a logarithmic scale, in such a way as to de-emphasize larger variations. The median of each participant’s five repeated measurements was computed for each loudspeaker position. It is reasonable to assume that adult listeners with NH responses are normally distributed because they are likely stable and consistent across positions [37]. The localization of a specific loudspeaker position in children who experienced auditory deprivation before being bilaterally implanted is less likely to follow a normal distribution [38]. Since our subsets often violated the assumption of a normal distribution of their residuals, linear mixed-effects models were not chosen for the analysis.

The analysis first compared each experimental variable of the two populations in the ON condition. Then, the other conditions were analyzed separately for each population. The data were hierarchical; they were aggregated via the median of all positions per participant and condition; moreover, they were divided by position and compared by condition. A Shapiro-Wilk test was performed to check if data residuals followed a normal distribution [39]. Given the limited sample size in some cases, the normality of the residuals was also tested with the Anderson-Darling test [40]; we did not report the results of the latter test because they rarely disagreed with the Shapiro-Wilk test results (14 cases over 504); when they did, we did not find statistical significance with any test of the subsequent analysis. If the samples came from the same population and the normality assumption was violated, a Friedman test [41] was performed, followed by a post-hoc Nemenyi test. The test employs the critical difference (CD) statistics and, according to Nemenyi [42], was developed to account for a family-wise error, hence being already a conservative test. For this reason, we did not apply *p*-adjustments. If the residuals from the same population followed a normal distribution, sphericity was checked with a Mauchly test [43]. If the sphericity hypothesis was met, we checked the equality of the means through an RM-ANOVA and performed a post-hoc analysis with a *t*-test; if sphericity was violated, the same tests were performed, but with a Greenhouse-Geisser correction of the *p*-value. If the samples came from two populations in the ON conditions, we checked the normality with a Shapiro-Wilk test and the homoscedasticity with a Levene test [44]. If the normality and homoscedasticity assumptions were met, we compared them with a *t*-test. If the latter was violated, a Welch *t*-test was employed. Otherwise, if evidence of a violation of normal distribution was found, a Mann-Whitney U test was used to compare the distribution with residuals with similar variances; diversely, a Yuen test [45] was used. The effect size was reported for every test; the partial *η*^2^ was computed for the repeated measures ANOVA and the Hedges’ *g* for the pairwise tests. An effect size (named *W*) was estimated as in Tomczak [46] for the non-parametric Friedman test. Hedges’ *g* was computed from Cohen’s *d* using an average variance if the samples came from the same population. A correlation analysis for each condition’s data was performed using Spearman’s *ρ* correlation coefficient since we did not assume a normal distribution of the variables. All tests were two-tailed. A Bonferroni correction was applied for every multiple comparison, except for the Nemenyi test. Statistical significance was set at *α* = 0.05. The analysis was made using the Python packages Pingouin [47] and Scipy [48] and the data visualization libraries Seaborn [49] and Matplotlib [50].

## Results

**Fig 2:**
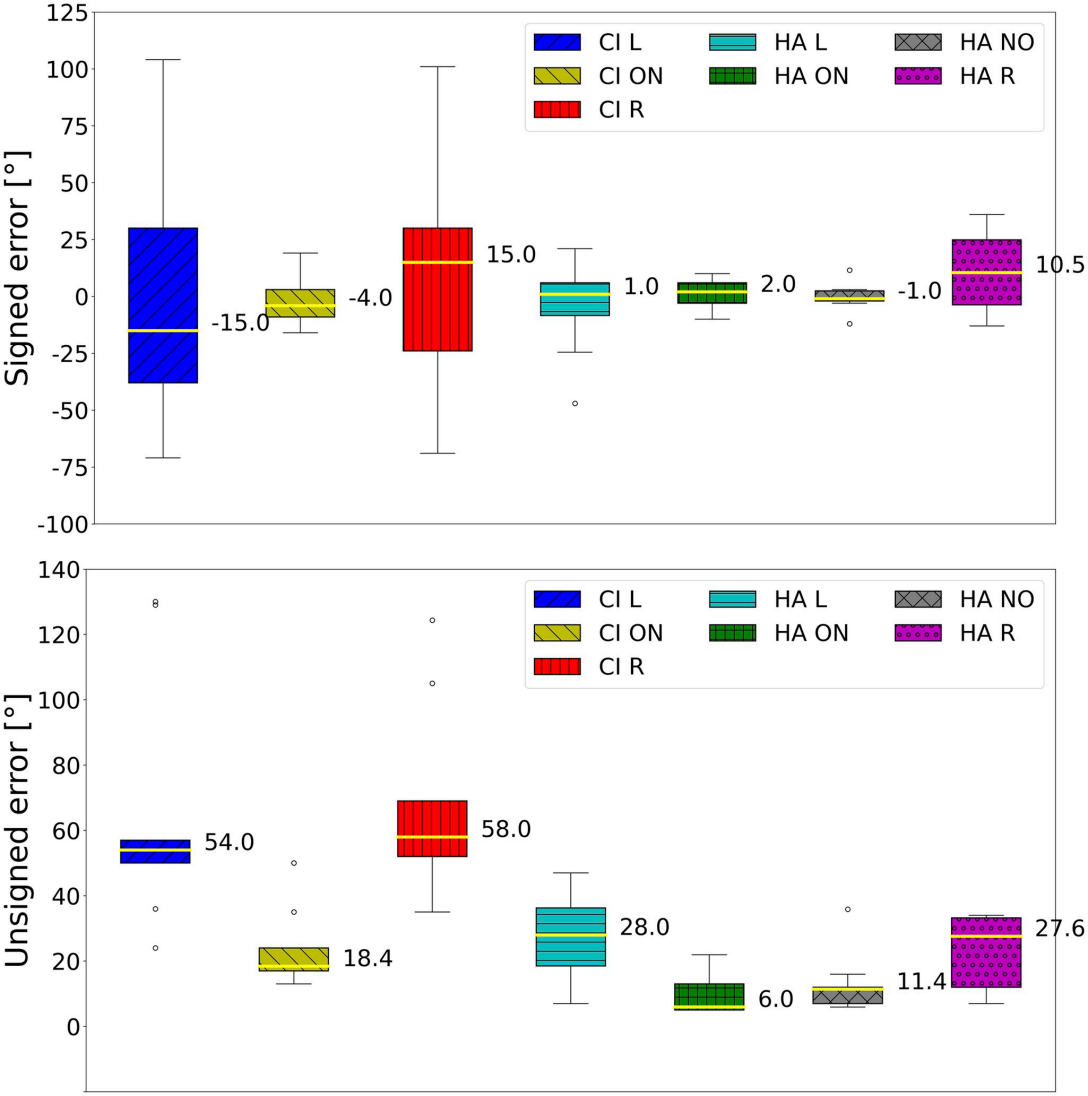
Medians across conditions of signed and unsigned errors.

**Fig 3:**
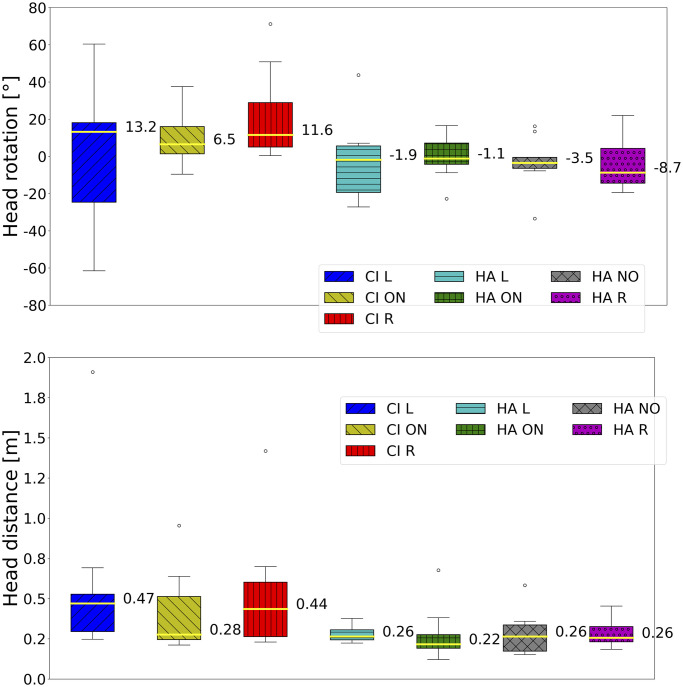
Medians across conditions of head rotation and head distance.

Medians of signed error, unsigned error, head rotation, and distance are graphically summarized in Figs 2 and 3 for each test condition, aggregated by target and participant. In the ON condition, the CI listeners’ median of the unsigned error was significantly worse than that of the HA listeners. The CI listeners’ medians of the signed error were the farthest from zero in the respective conditions. The signed error in the asymmetrical hearing conditions exhibited the largest variance. The second largest variance was found in the HA R condition, where the median was the third farthest from zero. The medians of the signed error in listeners with both HAs turned on and off were comparable to the mean performance of young NH listeners in the same mixed environment [24] (*μ* = 0.60°, *σ* = 5.26). The median of the unsigned error in HA listeners with both devices turned on was similar to the average performance of NH listeners [24] (*μ* = 4.15°, *σ* = 3.28); CI listeners did not fall within this range even in the CI ON condition. The asymmetric hearing condition increased the unsigned error of the former population by more than four times, while it increased for the latter population by three times. The accuracy in the HA NO condition was the second-best.

Fig 3 shows that asymmetrical hearing leads to the highest head rotation variances. HA listeners had the head rotation median closest to zero when both devices were turned on. Compared to HA listeners, the head distance covered by CI listeners in every condition had larger variances and medians. Moreover, even if the CI ON condition had a smaller median, the CI listeners’ head distance variances were similar. The HA ON condition exhibited the lowest head dynamism. Compared to NH listeners [24] (*μ* = 0.13 m), HA listeners (*μ* = 0.22 m) and CI listeners (*μ* = 0.28 m) increased head movement.

The statistical analysis first considered the differences between CI and HA populations in the bilateral listening condition and then the differences between conditions for each population separately. The results with statistical significance are presented in Table 2. The statistical analysis supported these observations: the accuracies of the two populations in the ON condition were different, and similarly were the head distances. Within each population, the ON condition led to significantly better accuracy than the asymmetric conditions, except for the HA R condition, which was not found to be statistically different.

**Table 2:** Comparison among medians of the variables across condition ON, conditions factorized to CI listeners and HA listeners. Only statistically significant results are illustrated.

Variable	Conditions	Normality	Sphericity	Homoscedasticity	Statistical test	Post-hoc analysis
Unsigned error	CI ON HA ON	CI ON, **True**: *W* = 0.90, *p* = 0.25 HA ON, **False**: *W* = 0.83, *p* = 0.18 * 10^−1^	-	**True**: *W* = 0.80, *p* = 0.38, *df* = (1, 20)	Mann-Whitney: *U* = 106.0, *p* = 0.16 * 10^−2^, *g* = 1.81	-
	CI L CI ON CI R	CI L, **True**: *W* = 0.89, *p* = 0.18 CI ON, **True**: *W* = 0.90, *p* = 0.25 CI R, **True**: *W* = 0.95, *p* = 0.64	**True**: *W* = 0.96, *p* = 0.87, *df* = 2	-	RM ANOVA: *F* = 21.97, *p* = 0.26 * 10^−4^, *df* = (2, 16), $\eta_{p}^{2}=0.73$	CI ON—CI L: *T* = −5.34, *p* = 0.21 * 10^−2^, *g* = −1.89 CI ON—CI R: *T* = −5.61, *p* = 0.15*10^−2^, *g* = −2.56
	HA L HA ON HA NO HA R	HA L, **True**: *W* = 0.86, *p* = 0.11 HA ON, **False**: *W* = 0.83, *p* = 0.18 * 10^−1^ HA NO, **True**: *W* = 0.89, *p* = 0.19 HA R, **False**: *W* = 0.81, *p* = 0.04	-	-	Friedman: *F* = 9.86, *p* = 0.20 * 10^−1^, *df* = 3, *W* = 0.21	HA L—HA ON: *CD* = 0.95, *p* = 0.46 * 10^−1^, *g* = 1.53
Head distance	CI ON HA ON	CI ON, **False**: *W* = 0.83, *p* = 0.46 * 10^−1^ HA ON, **False**: *W* = 0.79, *p* = 0.48 * 10^−2^	-	**True**: *W* = 1.30, *p* = 0.27, *df* = (1, 20)	Mann-Whitney: *U* = 89.0, *p* = 0.45 * 10^−1^, *g* = 0.77	-

**Fig 4:**
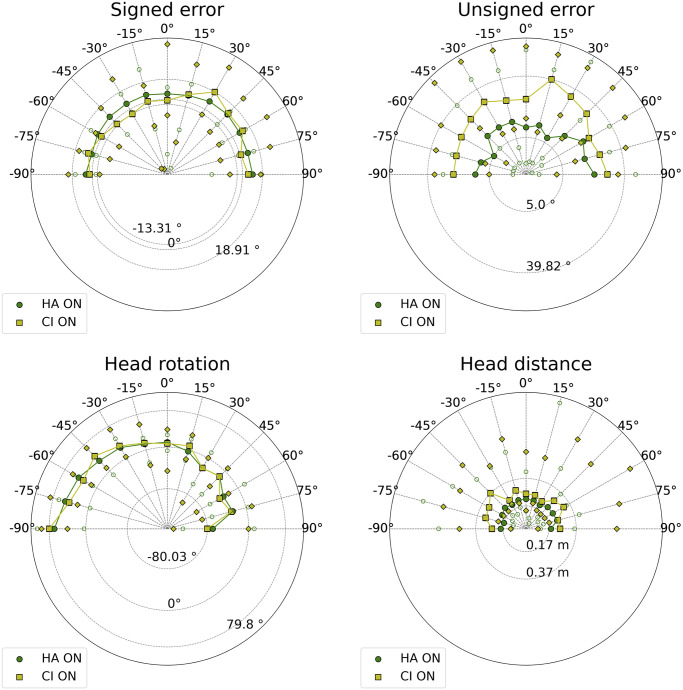
Signed errors, unsigned errors, head rotations, and distances of CI and HA listeners in the ON condition across positions. The data are normalized. Filled circles represent the HA ON medians, empty circles represent the HA ON minima and maxima. Squares represent the CI ON medians, and diamonds represent the CI ON minima and maxima.

The data of the two populations across loudspeaker positions in the ON condition are displayed in Fig 4, and the results having statistical significance are presented in Table 3. For eight targets out of thirteen, the HA listeners’ signed error variances were smaller than the CI listeners’, but only at −60° were the medians significantly different. The HA listeners’ unsigned error minima, medians, and maxima were always smaller than the corresponding CI listeners’ ones, except for the 30° and 90° maxima. The difference between the unsigned error’s medians of the two populations was always statistically significant, except for ±90°. The peak of the unsigned error of the CI listeners occurred at 15°, while the HA listeners’ one was located at 90°.

**Table 3:** Statistical results of the variables of CI and HA listeners in the ON condition across positions. Only statistically significant results are illustrated.

Variable	Target	Normality	Homoscedasticity	Statistical test
Signed error	−60°	CI ON, **True**: *W* = 0.91, *p* = 0.34 HA ON, **True**: *W* = 0.96, *p* = 0.81	**False**: *W* = 6.72, *p* = 0.17 * 10^−1^, *df* = (1, 20)	Welch: *T* = −2.34, *p* = 0.42 * 10^−1^, *g* = −1.13
Unsigned error	−75°	CI ON, **True**: *W* = 0.95, *p* = 0.65 HA ON, **True**: *W* = 0.97, *p* = 0.90	**True**: *W* = 0.04, *p* = 0.84, *df* = (1, 20)	*t*-test: *T* = 3.63, *p* = 0.20 * 10^−2^, *g* = 1.51
	−60°	CI ON, **True**: *W* = 0.93, *p* = 0.47 HA ON, **True**: *W* = 0.96, *p* = 0.69	**True**: *W* = 0.10, *p* = 0.75, *df* = (1, 20)	*t*-test: *T* = 2.83, *p* = 0.13 * 10^−1^, *g* = 1.23
	−45°	CI ON, **False**: *W* = 0.74, *p* = 0.44 * 10^−2^ HA ON, **True**: *W* = 0.93, *p* = 0.33	**True**: *W* = 0.13, *p* = 0.73, *df* = (1, 20)	Mann-Whitney: *U* = 97.0, *p* = 0.11 * 10^−1^, *g* = 1.14
	−30°	CI ON, **True**: *W* = 0.98, *p* = 0.94 HA ON, **True**: *W* = 0.99, *p* = 0.99	**True**: *W* = 0.21 * 10^−3^, *p* = 0.99, *df* = (1, 20)	*t*-test: *T* = 2.30, *p* = 0.03, *g* = 0.96
	−15°	CI ON, **True**: *W* = 0.84, *p* = 0.37 HA ON, **True**: *W* = 0.92, *p* = 0.25	**True**: *W* = 0.84, *p* = 0.37, *df* = (1, 20)	*t*-test: *T* = 2.19, *p* = 0.45 * 10^−1^, *g* = 0.95
	0°	CI ON, **False**: *W* = 0.83, *p* = 0.47 * 10^−1^ HA ON, **True**: *W* = 0.95, *p* = 0.54	**True**: *W* = 0.22, *p* = 0.65, *df* = (1, 20)	Mann-Whitney: *U* = 93.0, *p* = 0.23 * 10^−1^, *g* = 1.03
	15°	CI ON, **True**: *W* = 0.89, *p* = 0.21 HA ON, **True**: *W* = 0.93, *p* = 0.35	**True**: *W* = 0.13, *p* = 0.72, *df* = (1, 20)	*t*-test: *T* = 4.30, *p* = 0.44 * 10^−3^, *g* = 1.78
	30°	CI ON, **True**: *W* = 0.93, *p* = 0.51 HA ON, **True**: *W* = 0.90, *p* = 0.12	**True**: *W* = 0.99, *p* = 0.33, *df* = (1, 20)	*t*-test: *T* = 3.39, *p* = 0.30 * 10^−2^, *g* = 1.29
	45°	CI ON, **True**: *W* = 0.96, *p* = 0.80 HA ON, **True**: *W* = 0.96, *p* = 0.77	**True**: *W* = 0.37, *p* = 0.55, *df* = (1, 20)	*t*-test: *T* = 3.44, *p* = 0.40 * 10^−2^, *g* = 1.51
	60°	CI ON, **True**: *W* = 0.88, *p* = 0.16 HA ON, **True**: *W* = 0.93, *p* = 0.39	**True**: *W* = 0.01, *p* = 0.93, *df* = (1, 20)	*t*-test: *T* = 2.32, *p* = 0.32 * 10^−1^, *g* = 0.93
	75°	CI ON, **True**: *W* = 0.94, *p* = 0.63 HA ON, **True**: *W* = 0.95, *p* = 0.59	**True**: *W* = 0.44, *p* = 0.52, *df* = (1, 20)	*t*-test: *T* = 2.66, *p* = 0.15 * 10^−1^, *g* = 1.05
Head distance	−75°	CI ON, **False**: *W* = 0.77, *p* = 0.83 * 10^−2^ HA ON, **False**: *W* = 0.64, *p* = 0.15 * 10^−3^	**True**: *W* = 0.01, *p* = 0.93, *df* = (1, 20)	Mann-Whitney: *U* = 95.0, *p* = 0.16 * 10^−1^, *g* = 0.67
	−45°	CI ON, **True**: *W* = 0.93, *p* = 0.45 HA ON, **False**: *W* = 0.79, *p* = 0.59 * 10^−2^	**True**: *W* = 4.26, *p* = 0.52 * 10^−1^, *df* = (1, 20)	Mann-Whitney: *U* = 94.0, *p* = 0.19 * 10^−1^, *g* = 1.14
	−30°	CI ON, **False**: *W* = 0.80, *p* = 0.02 HA ON, **False**: *W* = 0.71, *p* = 0.70 * 10^−3^	**True**: *W* = 1.75, *p* = 0.20, *df* = (1, 20)	Mann-Whitney: *U* = 95.0, *p* = 0.16 * 10^−1^, *g* = 0.99

The HA listeners’ head distance medians were always smaller than the CI listeners’ ones, but only for three target positions were they significantly different.

**Fig 5:**
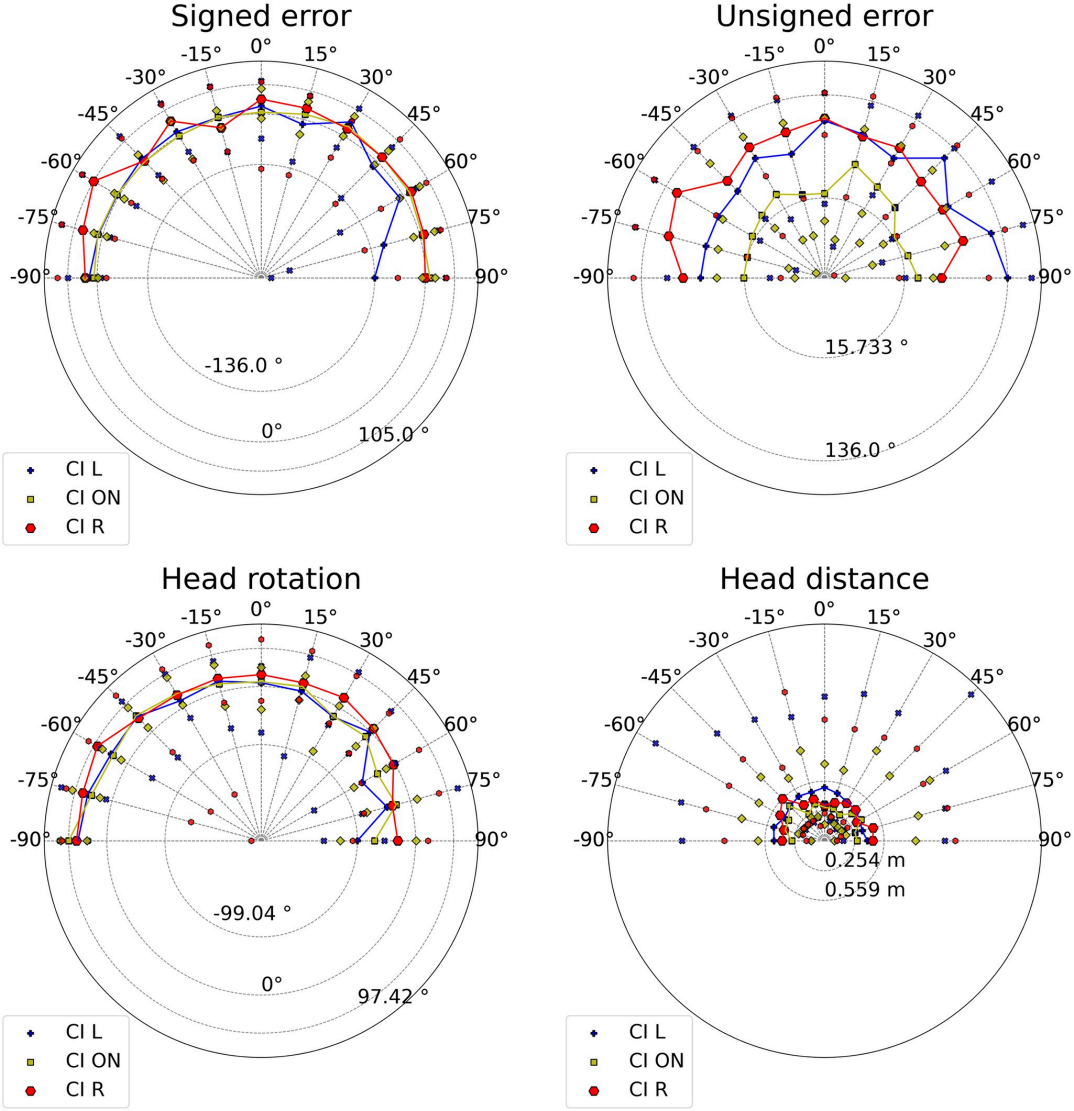
Signed errors, unsigned errors, head rotations, and distances of CI listeners in every condition across positions. The data are normalized. Circles represent the medians, triangles pointing down represent maxima, and triangles pointing up represent minima. Squares represent the CI ON medians, and diamonds represent the CI ON minima and maxima. Pluses represent the CI L medians, and xs represent the CI L minima and maxima. Hexagons represent the CI R medians. When rotated, they represent the CI R minima and maxima.

**Fig 6:**
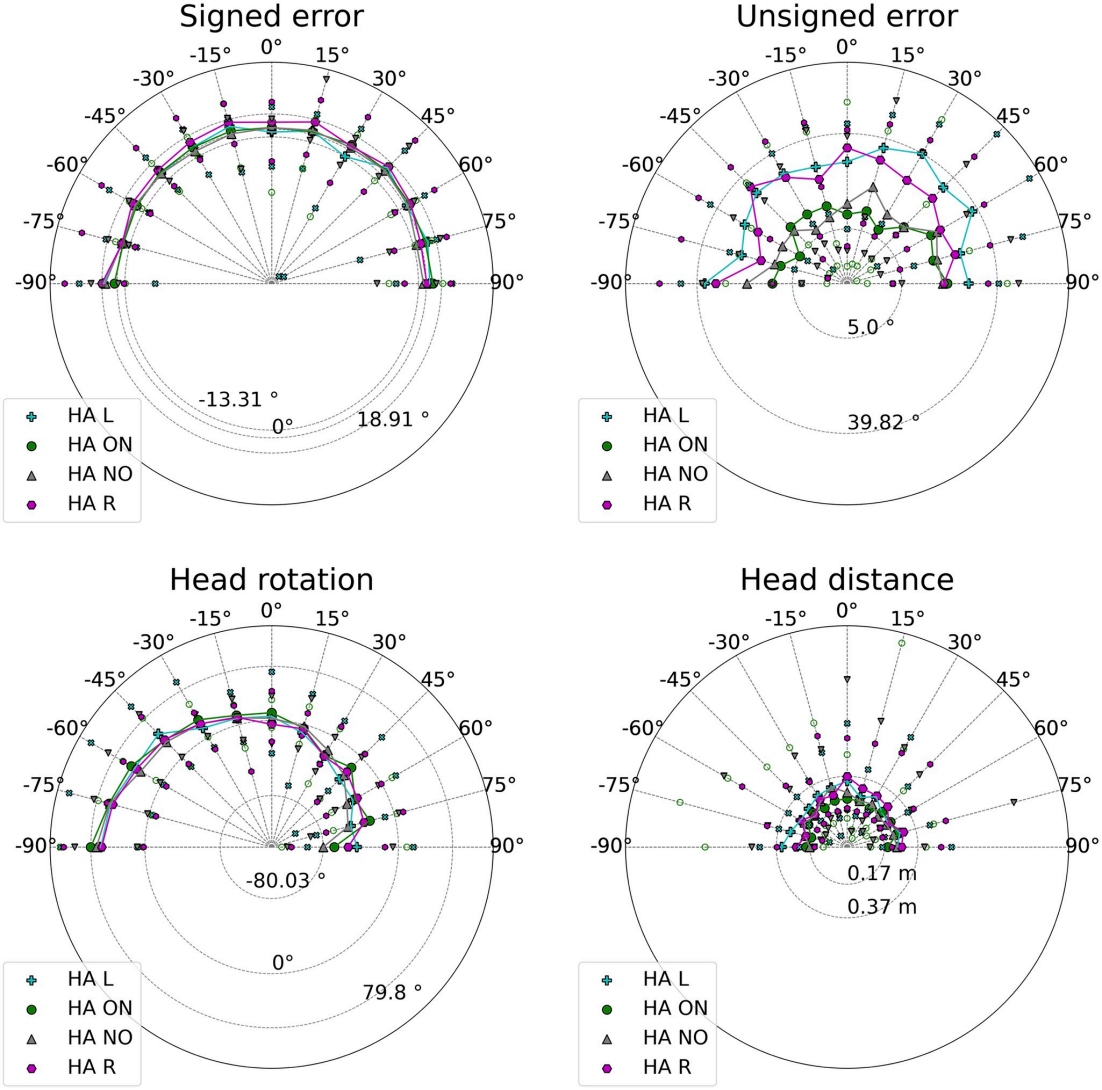
Signed errors, unsigned errors, head rotations, and distances of HA listeners in every condition across positions. The data are normalized. Filled circles represent the HA ON medians, and empty circles represent the HA ON minima and maxima. Pluses represent the HA L medians, and xs represent the HA L minima and maxima. Hexagons represent the HA R medians. When rotated, they represent the HA R minima and maxima. Triangles pointing up represent the HA NO medians, and triangles pointing up represent the HA NO minima and maxima.

The data divided by population in each condition are illustrated in Figs 5 and 6. The statistically significant results are shown in Tables 4 and 5. The asymmetrical hearing conditions worsened CI listeners’ accuracy at every target position, except for ±15°. CI R’s accuracy was significantly worse than CI ON’s everywhere, except for ±15°, 45°, 75°, and 90°. CI L’s accuracy was significantly worse than CI ON’s at −90°, 0°, and every target position in the right hemifield, except for 15°.

**Table 4:** Statistical results of the variables of CI listeners in every condition across positions. Only statistically significant results are illustrated.

Variable	Target	Normality	Sphericity	Statistical test	Post-hoc analysis
Signed error	75°	CI L, **True**: *W* = 0.95, *p* = 0.66 CI ON, **True**: *W* = 0.97, *p* = 0.89 CI R, **True**: *W* = 0.87, *p* = 0.12	**True**: *W* = 0.56, *p* = 0.13, *df* = 2	RM ANOVA: *F* = 4.26, *p* = 0.33 * 10^−1^, *df* = (2, 16), $\eta_{p}^{2}=0.35$	*p* > *α*
Unsigned error	−90°	CI L, **True**: *W* = 0.95, *p* = 0.69 CI ON, **True**: *W* = 0.90, *p* = 0.26 CI R, **True**: *W* = 0.94, *p* = 0.55	**True**: *W* = 0.48, *p* = 0.08, *df* = 2	RM ANOVA: *F* = 6.92, *p* = 0.68 * 10^−2^, *df* = (2, 16), $\eta_{p}^{2}=0.46$	CI ON—CI L: *T* = −4.00, *p* = 0.12 * 10^−1^, *g* = −1.43 CI ON—CI R: *T* = −3.91, *p* = 0.13 * 10^−1^, *g* = −1.42
	−75°	CI L, **True**: *W* = 0.94, *p* = 0.58 CI ON, **True**: *W* = 0.95, *p* = 0.65 CI R, **True**: *W* = 0.91, *p* = 0.35	**True**: *W* = 0.88, *p* = 0.63, *df* = 2	RM ANOVA: *F* = 11.95, *p* = 0.67 * 10^−3^, *df* = (2, 16), $\eta_{p}^{2}=0.60$	CI ON—CI R: *T* = −5.14, *p* = 0.27 * 10^−2^, *g* = −2.42
	−60°	CI L, **True**: *W* = 0.87, *p* = 0.12 CI ON, **True**: *W* = 0.93, *p* = 0.47 CI R, **True**: *W* = 0.91, *p* = 0.33	**True**: *W* = 0.82, *p* = 0.50, *df* = 2	RM ANOVA: *F* = 14.53, *p* = 0.25 * 10^−3^, *df* = (2, 16), $\eta_{p}^{2}=0.64$	CI ON—CI R: *T* = −6.70, *p* = 0.46 * 10^−3^, *g* = −2.81
	−45°	CI L, **True**: *W* = 0.93, *p* = 0.51 CI ON, **False**: *W* = 0.74, *p* = 0.44 * 10^−2^ CI R, **True**: *W* = 0.96, *p* = 0.84	-	Friedman: *F* = 6.22, *p* = 0.45 * 10^−1^, *df* = 2, *W* = 0.03	CI ON—CI R: *CD* = 0.95, *p* = 0.48 * 10^−1^, *g* = −1.18
	−30°	CI L, **True**: *W* = 0.96, *p* = 0.83 CI ON, **True**: *W* = 0.98, *p* = 0.94 CI R, **True**: *W* = 0.96, *p* = 0.80	**True**: *W* = 0.98, *p* = 0.93, *df* = 2	RM ANOVA: *F* = 5.92, *p* = 0.12 * 10^−1^, *df* = (2, 16), $\eta_{p}^{2}=0.44$	CI ON—CI R: *T* = −3.64, *p* = 0.20 * 10^−1^, *g* = −1.52
	−15°	CI L, **True**: *W* = 0.94, *p* = 0.53 CI ON, **True**: *W* = 0.89, *p* = 0.22 CI R, **True**: *W* = 0.91, *p* = 0.34	**True**: *W* = 0.89, *p* = 0.67, *df* = 2	RM ANOVA: *F* = 4.63, *p* = 0.26 * 10^−1^, *df* = (2, 16), $\eta_{p}^{2}=0.37$	*p* > *α*
	0°	CI L, **True**: *W* = 0.88, *p* = 0.17 CI ON, **False**: *W* = 0.83, *p* = 0.47 * 10^−1^ CI R, **True**: *W* = 0.90, *p* = 0.24	-	Friedman: *F* = 10.67, *p* = 0.26 * 10^−1^, *df* = 2, *W* = 0.05	CI ON—CI L: *CD* = 0.99, *p* = 0.13 * 10^−1^, *g* = −1.18 CI ON—CI R: *CD* = 0.99, *p* = 0.13 * 10^−1^, *g* = −2.14
	30°	CI L, **True**: *W* = 0.91, *p* = 0.33 CI ON, **True**: *W* = 0.93, *p* = 0.51 CI R, **True**: *W* = 0.98, *p* = 0.96	**True**: *W* = 0.73, *p* = 0.33, *df* = 2	RM ANOVA: *F* = 19.03, *p* = 0.59 * 10^−4^, *df* = (2, 16), $\eta_{p}^{2}=0.70$	CI ON—CI L: *T* = −4.59, *p* = 0.60 * 10^−2^, *g* = −1.88 CI ON—CI R: *T* = −4.91, *p* = 0.36 * 10^−2^, *g* = −1.88
	45°	CI L, **True**: *W* = 0.87, *p* = 0.11 CI ON, **True**: *W* = 0.96, *p* = 0.80 CI R, **True**: *W* = 0.96, *p* = 0.85	**True**: *W* = 0.92, *p* = 0.76, *df* = 2	RM ANOVA: *F* = 12.24, *p* = 0.60 * 10^−3^, *df* = (2, 16), $\eta_{p}^{2}=0.60$	CI ON—CI L: *T* = −5.02, *p* = 0.31 * 10^−2^, *g* = −1.93
	60°	CI L, **True**: *W* = 0.93, *p* = 0.47 CI ON, **True**: *W* = 0.88, *p* = 0.16 CI R, **True**: *W* = 0.92, *p* = 0.40	**True**: *W* = 0.55, *p* = 0.12, *df* = 2	RM ANOVA: *F* = 10.91, *p* = 0.10 * 10^−2^, *df* = (2, 16), $\eta_{p}^{2}=0.58$	CI ON—CI L: *T* = −3.35, *p* = 0.30 * 10^−1^, *g* = −1.38 CI ON—CI R: *T* = −4.09, *p* = 0.10 * 10^−1^, *g* = −1.67
	75°	CI L, **True**: *W* = 0.88, *p* = 0.17 CI ON, **True**: *W* = 0.94, *p* = 0.63 CI R, **True**: *W* = 0.89, *p* = 0.21	**True**: *W* = 0.90, *p* = 0.68, *df* = 2	RM ANOVA: *F* = 11.90, *p* = 0.68 * 10^−3^, *df* = (2, 16), $\eta_{p}^{2}=0.60$	CI ON—CI L: *T* = −5.15, *p* = 0.26 * 10^−2^, *g* = −1.75
	90°	CI L, **True**: *W* = 0.87, *p* = 0.12 CI ON, **False**: *W* = 0.82, *p* = 0.03 CI R, **True**: *W* = 0.93, *p* = 0.51	-	Friedman: *F* = 8.00, *p* = 0.18 * 10^−1^, *df* = 2, *W* = 0.04	CI ON—CI L: *CD* = 0.99, *p* = 0.13 * 10^−1^, *g* = −1.80
Head rotation	60°	CI L, **True**: *W* = 0.93, *p* = 0.46 CI ON, **True**: *W* = 0.91, *p* = 0.29 CI R, **True**: *W* = 0.92, *p* = 0.43	**True**: *W* = 0.65, *p* = 0.22, *df* = 2	RM ANOVA: *F* = 3.73, *p* = 0.47 * 10^−1^, *df* = (2, 16), $\eta_{p}^{2}=0.32$	*p* > *α*
	90°	CI L, **True**: *W* = 0.96, *p* = 0.81 CI ON, **True**: *W* = 0.92, *p* = 0.43 CI R, **True**: *W* = 0.93, *p* = 0.49	**True**: *W* = 0.99, *p* = 0.98, *df* = 2	RM ANOVA: *F* = 6.28, *p* = 0.97 * 10^−2^, *df* = (2, 16), $\eta_{p}^{2}=0.44$	CI L—CI R: *T* = −3.53, *p* = 0.23 * 10^−2^, *g* = −1.63
Head distance	0°	CI L, **False**: *W* = 0.77, *p* = 0.88 * 10^−2^ CI ON, **True**: *W* = 0.87, *p* = 0.12 CI R, **False**: *W* = 0.65, *p* = 0.40 * 10^−3^	-	Friedman: *F* = 11.56, *p* = 0.31 * 10^−2^, *df* = 2, *W* = 0.05	CI ON—CI L: *CD* = 1.00, *p* = 0.28 * 10^−2^, *g* = −0.93 CI L—CI R: *CD* = 0.95, *p* = 0.48 * 10^−1^, *g* = 0.49

**Table 5:** Statistical results of the variables of HA listeners in every condition across positions. Only statistically significant results are illustrated.

Variable	Target	Normality	Sphericity	Statistical test	Post-hoc analysis
Signed error	90°	HA L, **True**: *W* = 0.94, *p* = 0.59 HA ON, **False**: *W* = 0.82, *p* = 0.12 * 10^−1^ HA NO, **True**: *W* = 0.86, *p* = 0.10 HA R, **True**: *W* = 0.94, *p* = 0.56	-	Friedman: *F* = 8.10, *p* = 0.44 * 10^−1^, *df* = 2, *W* = 0.17	*p* > *α*
Unsigned error	−30°	HA L, **True**: *W* = 0.87, *p* = 0.15 HA ON, **True**: *W* = 0.99, *p* = 1.00 HA NO, **True**: *W* = 0.88, *p* = 0.15 HA R, **True**: *W* = 0.94, *p* = 0.61	**True**: *W* = 0.07, *p* = 0.54, *df* = 5 *df* = (3, 9), $\eta_{p}^{2}=0.57$	RM ANOVA: *F* = 3.96, *p* = 0.47 * 10^−1^,	*p* > *α*
	−15°	HA L, **True**: *W* = 0.96, *p* = 0.85 HA ON, **True**: *W* = 0.92, *p* = 0.25 HA NO, **True**: *W* = 0.91, *p* = 0.31	-	Friedman: *F* = 10.23, *p* = 0.17 * 10^−1^, *df* = 3, *W* = 0.21	HA R—HA ON: *CD* = 0.95, *p* = 0.46 * 10^−1^, *g* = 1.07
	15°	HA L, **True**: *W* = 0.91, *p* = 0.33 HA ON, **True**: *W* = 0.93, *p* = 0.35 HA NO, **True**: *W* = 0.88, *p* = 0.15 HA R, **True**: *W* = 0.94, *p* = 0.64	**True**: *W* = 0.00, *p* = 0.10, *df* = 5	RM ANOVA: *F* = 3.97, *p* = 0.47 * 10^−1^, *df* = (3, 9), $\eta_{p}^{2}=0.35$	*p* > *α*
	30°	HA L, **True**: *W* = 0.86, *p* = 0.12 HA ON, **True**: *W* = 0.90, *p* = 0.12 HA NO, **True**: *W* = 0.84, *p* = 0.05 HA R, **True**: *W* = 0.93, *p* = 0.49	**True**: *W* = 0.24, *p* = 0.82, *df* = 5	RM ANOVA: *F* = 5.68, *p* = 0.18 * 10^−1^, *df* = (3, 9), $\eta_{p}^{2}=0.65$	*p* > *α*
Head rotation	30°	HA L, **True**: *W* = 0.86, *p* = 0.12 HA ON, **True**: *W* = 0.97, *p* = 0.90 HA NO, **True**: *W* = 0.89, *p* = 0.19 HA R, **True**: *W* = 0.82, *p* = 0.05	**True**: *W* = 1.62 * 10^6^, *p* = 1.00, *df* = 5	RM ANOVA: *F* = 7.31, *p* = 0.87 * 10^−2^, *df* = (3, 9), $\eta_{p}^{2}=0.71$	HA NO—HA ON: *T* = −8.59, *p* = 0.20 * 10^−1^, *g* = −0.65
	75°	HA L, **True**: *W* = 0.95, *p* = 0.77 HA ON, **True**: *W* = 0.96, *p* = 0.76 HA NO, **True**: *W* = 0.96, *p* = 0.82 HA R, **True**: *W* = 0.93, *p* = 0.51	**True**: *W* = 8.40 * 10^1^, *p* = 1.00, *df* = 5	RM ANOVA: *F* = 4.09, *p* = 0.87 * 10^−2^, *df* = (3, 9), $\eta_{p}^{2}=0.58$	HA L—HA ON: *T* = −10.54, *p* = 0.11 * 10^−1^, *g* = −1.43

The asymmetric hearing led to larger unsigned error medians and maxima for the HA listeners; a significant difference was found only at −15° between the HA R and HA ON conditions. The HA listeners’ head distance maxima in asymmetrical hearing conditions were not always the largest, and medians were never significantly different.

**Fig 7:**
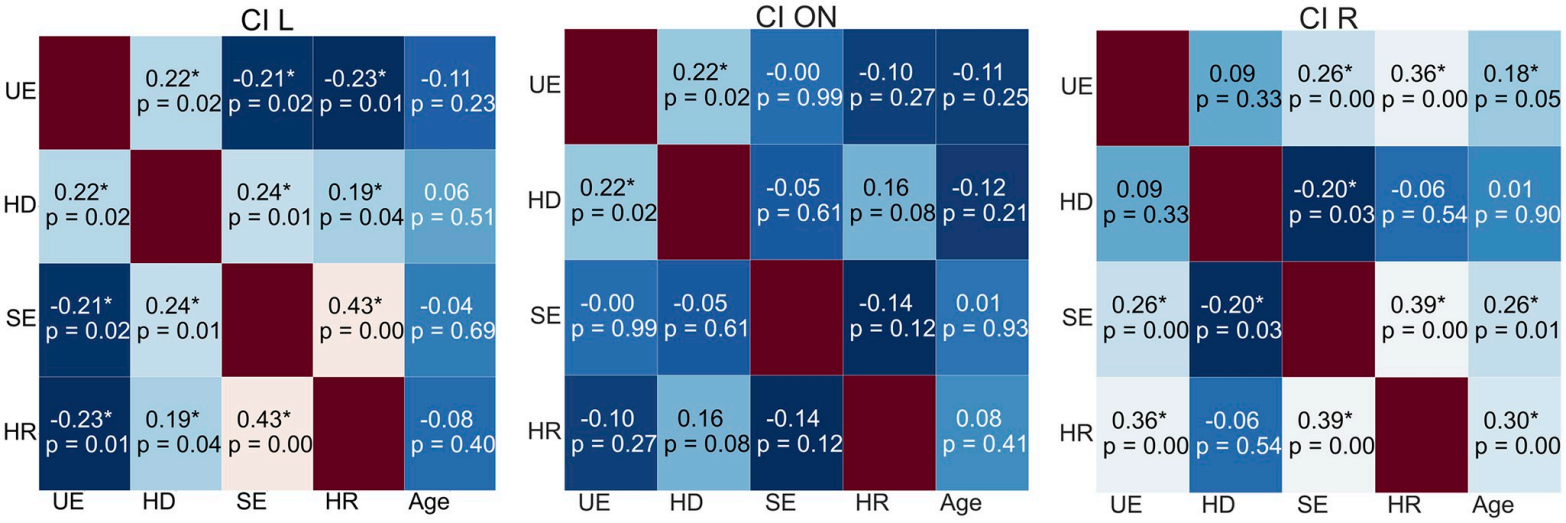
Heat maps reporting the correlations occurring in each condition of the CI listeners. Each asterisk indicates the statistical significance of the correlation. *UE* stands for unsigned error, *HD* for head distance, *SE* for signed error, and *HR* for head rotation.

**Fig 8:**
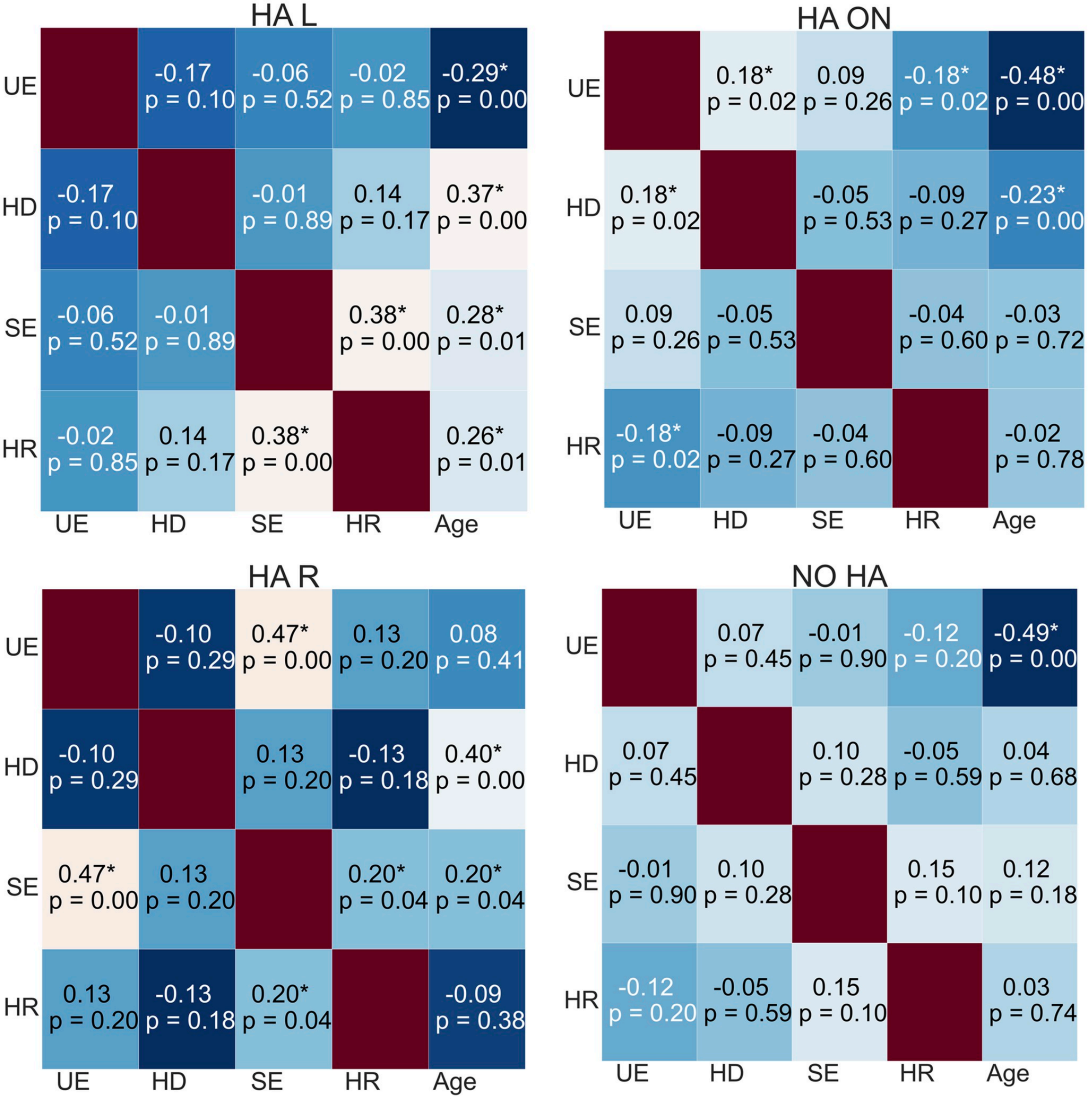
Heat maps reporting the correlations occurring in each condition of the HA listeners. Each asterisk indicates the statistical significance of the correlation. *UE* stands for unsigned error, *HD* for head distance, *SE* for signed error, and *HR* for head rotation.

The correlations between the experimental variables and individual age in every condition can be inspected from the heat maps in Figs 7 and 8.

When both CIs were turned on, the head distance correlated positively with the unsigned error. When only one CI was turned on, the head rotation and the signed error correlated with the unsigned error, positively when the left CI was turned off, and negatively when the right CI was turned off. In the asymmetrical conditions, the head distance correlated with the signed error positively when the right CI was turned off, and negatively when the left CI was turned off.

Considering the correlations in HA listeners, age correlated positively with accuracy in every condition, except when the left HA was turned off. Also for the HA listeners, head distance correlated positively with the unsigned error when both devices were turned on. Finally, age correlated negatively with the head distance when both devices were turned on, and positively in both the asymmetrical conditions.

## Discussion

Here, we summarize the main findings that emerged from the results:

- CI listeners did not process available localization cues as effectively as HA listeners did. With bilateral devices on, CI children performed worse than HA children across medians and overall, except for the most eccentric target locations.
- CI children were more dynamic than HA children with bilateral devices on. CI children’s head movements presented a large variance in every condition, indicating uncertainty and difficulty in localization. The correlation analysis suggested active listening did not improve CI children’s localization. Children with HAs exploited their residual and previous binaural ability in each hearing condition. Asymmetric hearing conditions impaired the performance of children with CIs much more than children with HAs.
- Children with HAs exploited their residual and previous binaural ability in each hearing condition. Asymmetric hearing conditions impaired the performance of children with CIs much more than that of children with HAs.
- CI children’s localization strategy was based on intensity cues. In asymmetric hearing conditions, CI listeners aimed to maximize the intensity at the aided ear; head rotation correlated with the accuracy, showing a clear tendency to turn that ear towards the sound source. Conversely, when the target was in the hemifield of the assisted ear, symmetric and monolateral conditions did not result in significantly different accuracy.
- Age correlated positively with accuracy in children with HAs in three hearing conditions. This correlation suggested that HA children acquired spatial hearing abilities through binaural experiences. Asymmetric hearing conditions affected head dynamism in older HA individuals; this sub-group, hence, likely made proficient use of active listening.

### Bilateral hearing: CI head movements and HA binaural skills

The bias and accuracy medians represented by the signed and unsigned error boxplots in Fig 2 ranked as expected among populations [51]. CI listeners’ accuracy with both CIs turned on (median: 18.4°) was consistent with previous research, where absolute azimuth errors in the frontal space did not exceed 39.4° [38, 52]. Nevertheless, our tests uncovered higher variances and numbers of outliers, probably because no visual cues were available to support localization, unlike previous reports that used a touch screen for response validation, and in which loudspeakers were visible [38, 52]. CI listeners’ accuracy was significantly worse than HA listeners’, both medians and across positions; the difference was not statistically significant at the most eccentric positions, where binaural ability was less necessary. Moreover, the CI listeners’ unsigned error peak was reached in correspondence with a central target position, i.e., 15°, where the effect size of the difference with the HA listeners’ unsigned error was the highest (*g* = 1.78). These results confirmed the poor ITD sensitivity of CI listeners [53, 54], probably due to two main reasons: the absence of auditory experience during an early critical period [55], and the pulse rate at which CIs operate [56]. CI processors generally run at fixed rates between 900 and 3700 pulses per second (pps) [57]; faster temporal sampling of speech envelopes might improve speech recognition in CI users. Nevertheless, even 600 pps could be “too fast for ITD,” given the poor performance of CI listeners at rates of 300 pps or above [56].

An inspection of bias and accuracy revealed that the signed and unsigned errors in HA listeners performing localization with both devices turned off were close to the ones made with the devices turned on: the medians of the accuracy were 11.4° and 6.0°, respectively. The benefit of HAs to spatial hearing was found to be significant in old adults, even if poorer than predicted [15], and was confirmed in children. However, it cannot be generalized as not being statistically significant. The median of the angular bias improved when both devices were turned off (−1.0°) rather than on (2.0°), even if not significantly. This result has already been found in some studies [58]. It is interpretable as a residual binaural sensitivity of HA listeners, who can achieve good localization performance without device support. The importance of a prelingual binaural experience is supported by the strong correlation between age and accuracy in HA listeners when both their devices were turned on or both are turned off. Older listeners consolidated a longer experience, and relied on it for spatial hearing; in the HA ON condition, these individuals did not rely on dynamic cues produced by head movements, as the negative correlation between the head distance and the age suggested.

Concerning motor activity during the task in the ON condition, the results in Table 2 about head distances show that CI listeners were especially active; the head distance covered by HA listeners was significantly smaller. The positive correlation between head distance and unsigned error in the CI ON, HA ON, and HA NO conditions can be interpreted as a sign of uncertainty in localization [59]. Mueller et al. [60] found that head movements disturbed localization, yet they instructed their participants to move or, conversely, keep their heads still before attending to specific listening conditions. Head movements were spontaneous during our task and may have been amplified when a target proved difficult to localize.

### Asymmetrical hearing: CI intensity maximization and HA active search compensatory strategies

The accuracy and angular bias of CI listeners were significantly impacted by asymmetric hearing. The former decreased significantly, while the variance of the latter soared when one CI was turned off. Both were indicators of uncertainty and difficulty in localization. Unsigned errors increased at every target location except for ±15°. Accuracy did not significantly deteriorate for a target positioned in the aided ear’s hemifield if the CI was turned off on the other side. These findings suggested that CI children’s localization was mostly based on intensity [16]; their strategies aimed at maximizing intensity in the aided ear. This hypothesis was supported by the significant correlation between head rotation and accuracy. When the assisted ear pointed towards the source, the intensity was at its maximum, and the best performances were obtained. As shown in Fig 2, angular bias leaned toward the aided ear in CI asymmetrical conditions. CI children’s head distance seemed to mitigate the asymmetrical intensity. Moreover, it correlated positively with the head rotation when only the left CI was turned on, indicating a search for the intensity peak at the left ear.

Even if the signed and unsigned error variances increased in both asymmetrical conditions, the effect of asymmetrical hearing on the accuracy of HA listeners was way more limited. The post-hoc analysis found significant differences only at 15° between the bilateral hearing condition and the condition with the left HA turned off, and between the medians of the former condition and the one with the right HA turned off. Again, these two findings suggested that having just one device turned on created confusion and increased difficulty in localization. HA listeners compensated for the adverse condition more efficiently; the difference in accuracy was found at an almost frontal target position. Here, binaural differences are more subtle, requiring greater sensitivity. Unlike CI listeners, HA children’s data in asymmetrical conditions did not present a correlation between head rotation and unsigned error. This indicated that they probably used residual binaurality for spatial hearing.

Fig 3 shows a general tendency for HA children to orient the right ear towards the source in every condition. An advantage of the right ear in auditory processing has been firmly established in decades of behavioral, electrophysiological, and neuroimaging research [61]. Even the correlation between head rotation and unsigned error in the condition with both HAs turned on can be read as an attitude to favor right-ear intensity maximization.

Bias and head rotation were positively correlated when only one device, CI or HA, was turned on, indicating a tendency to couple the pointing gesture with head rotation under asymmetric listening conditions. Our result extends the training-induced observed behavior of the NH [62] to the HI.

Previous research found no relationship between head dynamism and age in children with CIs [5], and our results support this. Children with HAs behaved differently. Head dynamism correlated negatively with age in the ON condition. HA listeners who developed binaural sensitivity behaved like the NH population, as they did not need to rely on dynamic cues elicited by head movement. They did so in asymmetric hearing conditions. Older HA listeners produced more pronounced head dynamism, as the positive correlation between head distance and age showed. They faced localization cue disruptions with dynamical changes in binaural cues, enabling a more reliable response in difficult hearing conditions [63]. The negative correlation between unsigned error and age confirmed that they were also the most successful in localization, at least when the right HA was turned off. Ultimately, the correlation with age suggests that active listening is refined throughout life [5].

These findings could be exploited when planning specific interventions for diverse HI pediatric populations. Children with HA must be assisted in acquiring binaural skills, not necessarily by insisting on head movement or unilateral maximization of intensity, unless they are in adverse hearing situations like speech-in-noise. The bilateral perception of children with CI should be trained, and vice versa, by insisting on active search motor behavior that includes unilateral training of even the weaker hemifield.

Spatial hearing investigations ask clinical research to take everyday listening into deeper consideration. The current study highlighted how crucial it is to support more ecological scenarios so that active listening can be taken into account when assessing hearing ability. An open research question is whether our results would be confirmed after the substitution of noise bursts with the most informative sound messages we are exposed to during the day, that is, speech. Although pink noise is a standard stimulus in the literature [24], Neuman et al. [51] did not find differences in localization accuracy of speech and pink noise sources, suggesting that the salient ILD cues are preserved by vocal messages. However, the processing algorithms that an auditory device applies to pink noise in terms of signal compression, noise reduction, and directional sensitivity remain generally unknown outside the manufacturing company. At any rate, we remain non-committal about the effectiveness of an accurate rendering of ITDs with CI because individuals who have not accumulated enough experience with these cues may not be responsive as expected.

## Conclusion

This study examined how children rehabilitated with bilateral hearing aids and bilateral cochlear implants localized sounds in the anterior horizontal field with concealed visual cues. Head movement and orientation were instrumental for spatial hearing, with different roles for the two populations. Asymmetrical hearing causes the largest errors, particularly for cochlear implant users. Children with bilateral cochlear implants showed more active listening than children with bilateral hearing aids; nevertheless, their activity revealed uncertainty rather than configuring as an additional resource. In the latter population, a significant correlation was found between age and localization accuracy and between age and head movement. Listeners with hearing aids and a longer binaural hearing experience actively searched the sound source to face the disrupted binaural cues introduced by asymmetric hearing conditions. The dominance of intensity cues was confirmed for the population with cochlear implants.

Hints regarding strategies based on level maximization at the better hearing ear have been found in asymmetric listening conditions. The different reactions of the two populations to the adverse conditions introduced by asymmetric hearing were analyzed. They revealed that children with hearing aids can rely on richer binaural cues, localize through dynamic information, and sharpen this ability over time. A quantitative analysis of active listening may pave the way for new methodologies in auditory localization studies. They could objectively characterize the listeners’ spatial listening strategies based on their motor behavior in an ecological acoustic environment. Furthermore, based on the positioning of the head-mounted devices and their orientation angle when the target was hit, the data acquired during a test session might be used to train dynamic and adaptive algorithms enhancing the directionality of cochlear implants and hearing aids.
